# Conceptualizing the Shelter and Housing Needs and Solutions of Older People Experiencing Homelessness

**DOI:** 10.1093/geroni/igaa057.2488

**Published:** 2020-12-16

**Authors:** Sarah Canham, Joe Humphries

**Affiliations:** 1 University of Utah, Salt Lake City, Utah, United States; 2 Simon Fraser University, Vancouver, British Columbia, Canada

## Abstract

Newly and chronically homeless older adults have unique pathways into homelessness and distinct physical, mental, and social needs. Using a five-step process, we conducted a scoping review of primary research to investigate the needs and solutions for sheltering/housing older people experiencing homelessness (OPEH). Thematic analysis of data from 19 sources revealed 1) shelter/housing needs and challenges of newly vs. chronically homeless older adults; 2) existing shelter/housing solutions addressing the needs of OPEH, including Housing First, permanent supportive housing, and multiservice homelessness intervention programs; and 3) outcomes of rehousing OPEH. Following, we developed a conceptual model which outlines how unique health and psychosocial needs of newly and chronically homeless older adults can be met through appropriately-designed shelter/housing solutions with individualized levels of senior-specific support. Future shelter/housing initiatives and strategies should use a rights-based approach and prioritize matching diverse OPEH needs to appropriate shelter/housing options that will support their ability to age-in-the-right-place. Part of a symposium sponsored by the Environmental Gerontology Interest Group.

